# Estimating the surface area of birds: using the homing pigeon (*Columba livia*) as a model

**DOI:** 10.1242/bio.20146999

**Published:** 2014-05-08

**Authors:** Cristina R. Perez, John K. Moye, Chris A. Pritsos

**Affiliations:** Department of Agriculture, Nutrition and Veterinary Sciences, University of Nevada, Reno, NV 89557, USA

**Keywords:** Surface area, Homing pigeon, *Columba livia*

## Abstract

Estimation of the surface area of the avian body is valuable for thermoregulation and metabolism studies as well as for assessing exposure to oil and other surface-active organic pollutants from a spill. The use of frozen carcasses for surface area estimations prevents the ability to modify the posture of the bird. The surface area of six live homing pigeons in the fully extended flight position was estimated using a noninvasive method. An equation was derived to estimate the total surface area of a pigeon based on its body weight. A pigeon's surface area in the fully extended flight position is approximately 4 times larger than the surface area of a pigeon in the perching position. The surface area of a bird is dependent on its physical position, and, therefore, the fully extended flight position exhibits the maximum area of a bird and should be considered the true surface area of a bird.

## INTRODUCTION

Estimating the surface area of the body has been experimentally done on several species of animals including dogs, birds, cats, chickens, monkeys, and birds (Cowgill and Drabkin, 1927; Dawson, 1967; Hori et al., 1972; Lee and Fox, 1933; Mitchell, 1930; Stitt et al., 1971; Vaughan and Adams, 1967; Walsberg and King, 1978). Several of these studies have experimentally estimated the surface area in order to understand the thermal relations between an animal and its environment (Hori et al., 1972; Stitt et al., 1971; Vaughan and Adams, 1967; Walsberg and King, 1978), as well as for the use in examining the relationship between surface area and metabolic rate (Cowgill and Drabkin, 1927; Dawson, 1967; Lee and Fox, 1933; Mitchell, 1930). The methodology used is variable across studies depending on the species, available resources, and research objective. Many studies have used animal carcasses in order to allow for skinning or the application of material to wrap closely to the skin surfaces (Cowgill and Drabkin, 1927; Dawson, 1967; Hori et al., 1972; Lee and Fox, 1933; Mitchell, 1930; Stitt et al., 1971; Vaughan and Adams, 1967; Walsberg and King, 1978). However, using carcasses requires the need for dead animals, and prevents the ability to modify the physical position of the animal. In this study, we develop a method for noninvasively measuring the flight surface area of a bird using live homing pigeons (*Columba livia*).

## RESULTS AND DISCUSSION

**Fig. 1.**
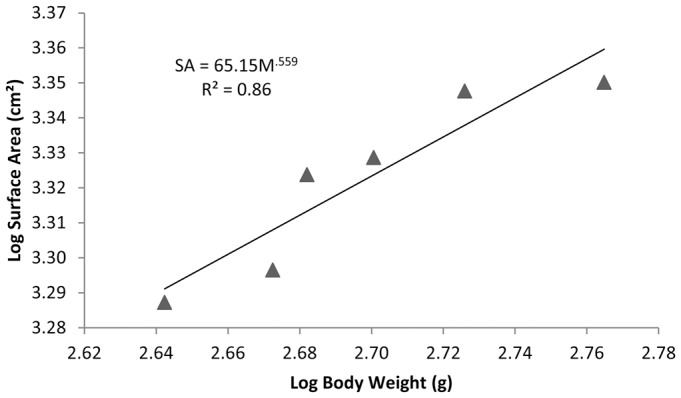
Log transformed relationship of total surface area to body weight for six homing pigeons. Solid line represents the linear regression equation where SA is the total bird surface area in cm^2^ and M is bird mass in g.

The measured regional and total surface areas for each bird are shown in Table 1. Table 1 also shows the proportion of each region to the total surface area. Irrespective of body weights, the regional proportionalities across the six birds is relatively consistent (Table 1). Across all body weights, the wing area makes up the majority of the total body surface area (58.9±1.78%) and the approximated surface area of the head constitutes the minority of the total body surface area (1.30±0.20%), and could be considered negligible. There is a significant positive correlation between body weight and body surface area (Pearson correlation test; r = 0.921, p = 0.009). The consistency of the regional proportionalities across varying body weights and significant positive correlation between body weight and total surface area suggest that as body weight increases, the total surface area increases, but the proportion of each regional area to the total surface area remains constant. The data were log transformed and plotted with a best fit linear regression line (Fig. 1). The relationship between body weight and body surface area shows a closely fitted positive linear relationship (R^2^ = 0.86). Because of the proportional relationship between body weight and body surface area, we can derive an equation for calculating the total surface area of a pigeon of known body weight.


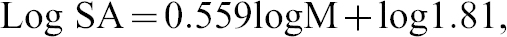
(1)
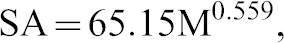
(2)where the surface area (SA) is in cm^2^ and the mass of the bird (M) is in grams. The average percent difference between the measured total surface area and the calculated total surface area using the derived equation (Eqn 2) for the 6 birds used in these studies was 1.92±0.72% S.D., with individual percent differences being less than 3%. The small percent difference signifies a close fit of the prediction formula to the surface area measurements of a pigeon.

**Table 1.**
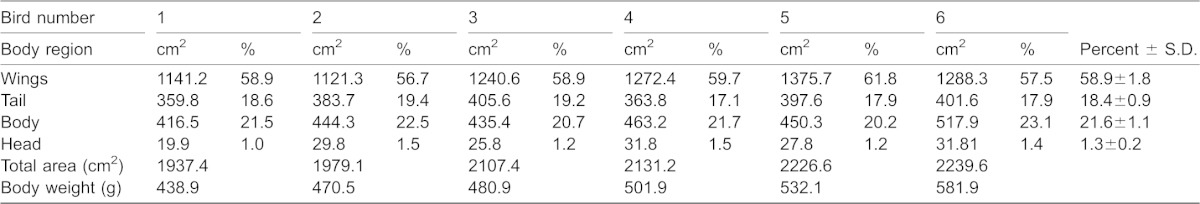
Body surface areas, regional proportionalities, and weights of six homing pigeons

In 1978, Walsberg and King developed a similar method for estimating the external surface area of birds (Walsberg and King, 1978). In their study, they used the measured surface areas of 8 different avian species to derive a relationship between the external body surface area and the weight of a bird as a derivative of Meeh's formula (Meeh, 1879). However, in their study, they used frozen bird carcasses and consequently the bird's surface area was measured solely in the perching position and excluded the area of the tail feathers and extended wings. Estimating the body surface area of a bird in the perching position only, where the area of the extended wings and tail is excluded, will highly underestimate the bird's surface area. As shown in Table 1, on average, the full extension of the wings makes up approximately 59% of the total surface area, the full extension of the tail makes up about 18% of the total surface area, while the body only comprises approximately 22% of the total surface area of a pigeon. In our study, the summation of the head and body regional surface areas would be equivalent to the total external surface area found in Walsberg and King. They found that for a 649.1 g pigeon, the total external surface area is 598.0 cm^2^ (Walsberg and King, 1978). However, using our equation (Eqn 2), we would calculate that a 649.1 g pigeon would have a surface area of approximately 2432.2 cm^2^, which is about a factor of 4 larger than that found by Walsberg and King. This suggests that in order to more accurately capture the true surface area of a bird, the physical body position must be taken into account.

Estimating a bird's surface area is important in studies where the bird's body size must be known, including thermoregulation, metabolism, flight aerodynamics, physiology, morphology, and growth studies (Walsberg and King, 1978; Thomas, 1996). It is also very important to know the surface area of a bird when assessing the level of exposure to environmental contaminants such as oil. The ability to accurately assess the percentage of surface area coverage by the contaminant may provide insights into the biological impact of that exposure, and thus the entire avian surface area must be considered, including that of the extended wings and tail. However, all species of birds exhibit different proportionalities of features, and thus creating a universal surface area equation for birds is difficult. In this study, we found wing area to be the most dominant factor in the estimation of total surface area. The shape and size of a bird's wing varies across avian species because they are heavily impacted by the selective pressures of migration, habitat, sexual selection, foraging behavior and predation (Copete et al., 1999). Flight is a characteristic adaptation of birds, and therefore the position of a bird's body while in flight is extremely important. In flight, birds have the ability to change their wing span, wing area, and tail spread in order to efficiently adjust the power needed during the changing dynamics of flight (Thomas, 1996). At low speed, wing span and tail spread should be at their maximum (Thomas, 1996). Thus, the maximum area of a bird would be at this flight position, and therefore should be considered the actual surface area of a bird. The equation derived here (Eqn 2) can be used to estimate this surface area of a pigeon based on its body weight. It can also be used to approximate the surface area of a bird exhibiting similar regional proportionalities as a pigeon. The methods described in this paper can be repeated for any species of bird to estimate the surface area in relation to body weight for that species. We conclude that the methodology for measuring the surface area of a bird presented in this study would more accurately estimate the true surface area and body size of a bird than previous attempts.

In addressing the field of thermoregulation, we are aware that excluding the surface area of the bill and legs excludes areas where significant heat transfer occurs. However, the degree of evaporative heat lost by the bill is dependent on the angle of the bill opening and not on the surface area of the bill itself. The cooling ability of the legs is dependent on the bird's decision to drop them during flight and allowing them to trail in the air stream. However, trailing the legs increases drag; consequently, decreasing flight efficiency and high levels of evaporative heat loss could result in dehydration. Therefore, these cooling techniques are largely used in extreme conditions and are not used regularly by passerine birds during flight at moderate ambient temperatures (Ward et al., 1999). Thus, the actual surface areas of the bill and the legs themselves are of minimal importance for flight thermoregulation. Ward et al. found that due to their large surface area, the wings of starlings in flight dissipate 55% of the overall heat loss through convection, despite the wings being among the coolest parts of the body (Ward et al., 1999). This suggests that the surface area of the extended wings plays a significant role in flight thermoregulation, contributing to the overall importance of including the surface area of the extended wings when calculating a bird's total surface area.

## MATERIALS AND METHODS

The pigeons used in this study were housed at the homing pigeon research field lab located at the Agricultural Experiment Station at the University of Nevada, Reno. All birds were maintained under an approved protocol by the university's animal care and use committee. Before morning feeding, six live birds were chosen according to their size in order to include a wide range of body weights. Each bird was weighed to the 0.01 g accuracy with an electronic balance before surface area measurements were taken. The area of the bird was divided into four different regions; head, tail, body, and wing. In this study we excluded the areas of the beak and legs as they only make up a small percentage of the total surface area (Walsberg and King, 1978). After being weighed, each bird was laid on a flat table surface that was overlaid with polyethylene film. The wing and the tail were laid out flat on the film so that the feathers of each region were completely spread. The shapes of these regions were traced with a marker onto the film. The body was defined as the base of the neck to the base of the tail feathers. The bird was placed on the film with its back flat on the surface. With the wings and legs compressed to the body as much as possible, the film was wrapped around the bird and the dimensions were marked. The head was laid sideways with the left ear down and the profile from the base of the beak to the base of the neck was traced onto the film. The surface area of each region was determined by tracing the polyethylene film regions onto paper of known weight per area and weighing the paper. The following adjustments to the regional surface areas were made in order to encompass the complete surface area. The surface area of the head was multiplied by two to approximate the whole head area. The surface area for the tail was multiplied by two to account for the ventral and dorsal side of the region. The surface area for the wing was multiplied by two to account for the ventral and dorsal side of the region and then multiplied by two again to account for both wings. The surface area of the body required no adjustments. The total body surface area was determined by the summation of the total surface areas for each region. The accuracy of this method was supported by geometrically measuring the area of the body region, which exhibits a rectangular shape. The average percent difference between the weighed paper area and the geometrically measured area was less than 5% (4.46±1.32% S.D.). The relationship between body weight and body surface area was analyzed by linear regression and Pearson's correlation test.
